# Assessment of Spine Patient Preferences for the Location of Surgery Between a Hospital and an Ambulatory Surgical Center in the Time of COVID-19: An Analysis of Patient Surveys

**DOI:** 10.7759/cureus.31655

**Published:** 2022-11-18

**Authors:** Nicholas Cassimatis, Geoffrey O'Malley, Emmanuel Ihionkhan, Roy Vingan, Mohammed F Khan, Hooman Azmi, Reza Karimi, Patrick A Roth

**Affiliations:** 1 Neurological Surgery, Hackensack University Medical Center, Hackensack, USA; 2 Neurological Surgery, New Jersey Brain and Spine, Oradell, USA

**Keywords:** patient preference, neurosurgery, ambulatory surgery center, covid 19, spine

## Abstract

Introduction

There has been a recent increase in the number of spinal procedures that can be performed in ambulatory surgical centers (ASCs). Studies have found that patients who undergo procedures at ASCs tend to have lower complication rates following procedures, including lower infection rates. Furthermore, ASCs offer significantly lower costs of procedures to patients and health insurance companies as compared to the costs of procedures performed in a hospital. Despite precautions and screening in place by ASCs, patients may be hesitant to undergo procedures outside of the hospital. Conversely, the ongoing COVID-19 pandemic has created hesitancy for many to go to the hospital for care due to the presence of COVID patients.

Objective

To assess patient preferences in the location of elective spine procedures between ASCs and hospitals, the authors conducted a survey of spine surgery candidates in a single practice.

Methods

A survey measuring patient age, vaccination status, fear of contracting COVID-19, and preference of surgery location was given to spinal surgery candidates at a single practice between fall 2021 and winter 2022. Statistical differences between the means of response groups were measured by a two-sample Z-score test.

Results

A total of 58 surveys were completed by patients. No difference in preference was observed by age. A difference was observed between genders, with 66% of females preferring ASCs to 40% of males (α=0.03). Patients with a fear of contracting COVID-19 preferred to have their procedure performed in an ASC. No difference was observed in location due to vaccination status, but unvaccinated patients had a significantly lower fear of contracting COVID-19 (α=0.02).

Conclusion

The differences in patient preferences have no clear cause, highlighting the need for better patient education in regard to the risks and benefits of each location of surgery. The fear of contracting COVID-19 on the day of surgery appears to be more ideological than rational for unvaccinated patients, who had less fear of contracting COVID-19 than vaccinated patients, despite being more likely to contract COVID-19 than vaccinated patients.

## Introduction

Recently, there has been an increase in the number of spinal procedures that can be performed in ambulatory surgical centers (ASCs) [1]. Relative to hospitals, ASCs offer patients quick and flexible scheduling of procedures. On the day of surgery, patients undergoing procedures in ASCs have a more personalized experience with a higher nurse-to-patient ratio than in a hospital. The higher nurse-to-patient ratio further allows ASCs to offer patients superior discharge times to home [2]. 

Studies have found that patients who undergo spine procedures at ASCs tend to have lower complication rates following procedures, including lower infection rates [3,4,5]. Furthermore, ASCs offer significantly lower costs of procedures to patients and health insurance companies than if the procedure was performed in a hospital [6-9]. The lower costs of ASCs offer a method to combat the ever-increasing costs of healthcare [10].

Procedures able to be performed in ASCs include but are not limited to lumbar laminectomies and discectomies. The future scope of procedures in ASCs is widening as transitioning of one and two-level lumbar and cervical fusion operations from hospitals to ASCs has also begun [1,11,12]. 

Despite the many clear advantages for patients having a procedure in an ASC compared to in a hospital, ASCs are not a viable option for all patients and procedures. Limits remain in regard to what patients can be selected to undergo procedures in ASCs. Patients typically excluded from undergoing procedures in ASCs are those with multiple comorbidities and those of advanced age [2]. Despite precautions and screening in place by ASCs, patients may also be hesitant to undergo procedures outside of the hospital [13]. Conversely, the ongoing COVID-19 pandemic has created hesitance for many to go to the hospital for care due to the presence of COVID patients [14]. 

To assess patient preferences in regard to the location of procedures between ASCs and hospitals, the authors conducted a survey of elective spine surgery candidates in a single practice.

## Materials and methods

Surveys were given to candidates for elective lumbar and cervical spinal surgery between September 2021 and February 2022. Patients were given the anonymous survey if they met the inclusion criteria as follows: (i) the patient was willing to participate, and (ii) the patient must be considering and/or being evaluated for the possibility of elective surgery. Patients were excluded from the study if they met the exclusion criteria as follows: (i) emergent surgical patients, (ii) non-English speakers/readers, (iii) mentally disabled/incompetent patients, or (iv) already completed the survey.

A total of 58 surveys were completed by patients. The surveys measured patient age, gender, vaccination status, fear of contracting COVID-19, and preference of surgical location. Vaccination status was measured by a response of "yes" or "no" to having received two doses of the Pfizer or Moderna vaccines or one dose of the Johnson & Johnson Vaccine. Fear of contracting COVID-19 was measured in the settings of completing activities of daily living (such as getting groceries), in medical offices, and in a hospital setting. Patients' fear of contracting COVID-19 was measured by rating their fear between one and five, with one being no fear of contracting COVID-19 and five representing being very fearful of contracting COVID-19. Patients were labeled as "high fear" in a particular category if they responded with fear greater than three. 

The patient preference for the location of surgery between an ambulatory surgical center and a hospital was prefaced with information regarding the types of procedures that could be performed in each location. Patients were informed of the limits on the types of patients that ambulatory surgical centers could accommodate due to co-morbidities and age, and the pros and cons of each choice. Results of the survey were collated, and simple statistics were run. Statistical differences between the means of response groups were measured by a two-sample Z-score test. A p-value of less than or equal to α=0.05 was considered statistically significant for all tests. All the analyses were performed using Microsoft Excel (Microsoft Corporation, Redmond, Washington).

## Results

**Figure 1:**
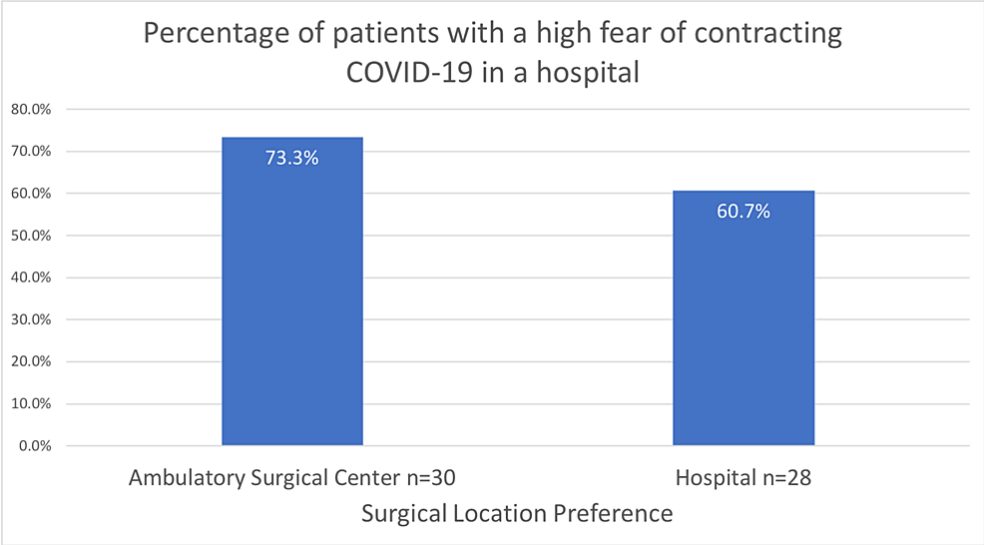
Percentage of patients with high fear of contracting COVID-19 in a hospital

A total of 58 surveys were completed by patients. Thirty patients responded they would prefer to have their operation at the ambulatory surgical center, while 28 responded they would prefer to have their surgery at the hospital. The patients surveyed had a range of ages between 25 and 91, with the average being 51. Between preferences of surgical location, no difference was observed in regards to age, vaccination status, and fear of contracting COVID-19 in performing activities of daily living and in healthcare settings (Table 1). The patients who preferred the ambulatory surgical center had a range of ages from 28 to 88, while the group that preferred the hospital had a range of ages from 25 to 91. A higher percentage of patients who preferred to have their procedures at the ambulatory surgical center had high fear of contracting COVID-19 in a hospital (73.3% vs. 60.7%, Figure 1). 

**Table 1:** Surgical location preference data

Surgical location preference	Ambulatory surgical center n=30		Hospital n=28		
Response	Raw/average	Percent/SD	Raw/average	Percent/SD	p-value
Age	49.73	16.81	52.68	18.16	0.522
Male	12	40.0%	18	64.3%	0.044
Vaccinated against COVID-19	19	63.3%	18	64.3%	0.936
High fear of contracting COVID-19 getting groceries, running errands, etc.	15	50.0%	14	50.0%	1
High fear of contracting COVID-19 in a healthcare office	16	53.3%	15	53.6%	0.984
High fear of contracting COVID-19 in a hospital	22	73.3%	17	60.7%	0.308

**Figure 2:**
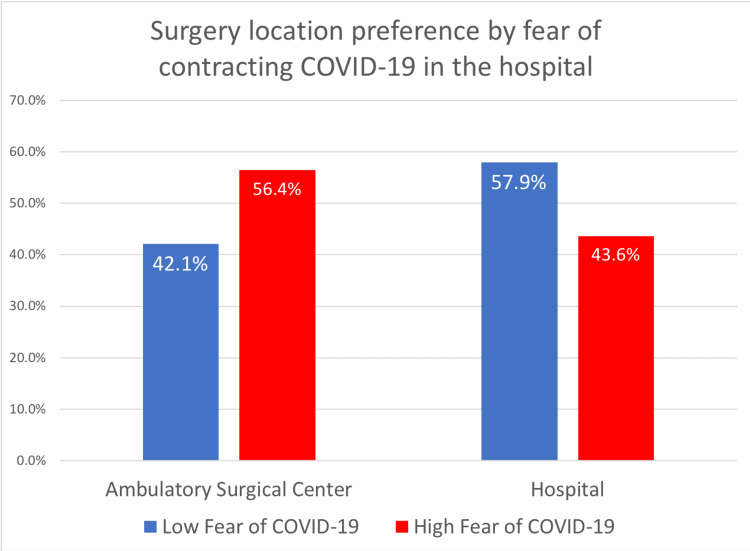
Surgery location preference by fear of contracting COVID-19 in the hospital

Furthermore, when comparing patients' preferences by the level of fear of contracting COVID-19 in a hospital setting, patients with high fear continue to prefer their procedures to take place in an ambulatory surgical center (Figure 2). Patients with high fear of contracting COVID-19 in a hospital setting tended to be older (55 vs. 43, α=0.00), female (53% vs. 41%, α=0.11), and had higher rates of vaccination against COVID-19 (76% vs. 42%, α=0.00). 

**Figure 3:**
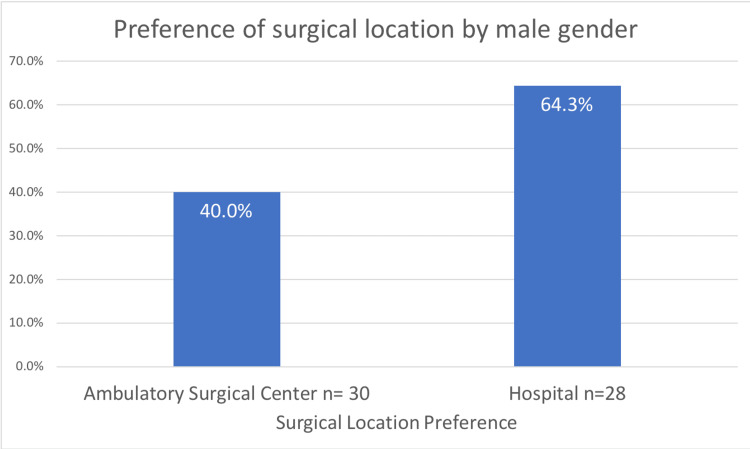
Preference of surgical location by the male gender

Moreover, a larger group of males preferred to undergo procedures at the hospital compared to the ambulatory surgical center (64.3% vs. 40%, α=0.04, Figure 3). This occurs with males tending to have lower rates of high fear of contracting COVID-19 in the hospital as compared to females (41% vs. 53%). Additionally, males reported lower rates of vaccination than females did (40% vs. 55%).

## Discussion

The findings of the patient preference surveys yield some expected results but also some unexpected results. The surveys confirm a relatively intuitive tendency of patients who fear contracting COVID-19 usually prefer to have elective spine operations in an ambulatory surgery center. Those less fearful of COVID-19 seem to prefer the added security and patient safety offered in a hospital setting. It is expected that patients less fearful of infection by COVID-19 will be more likely to value the availability of hospital resources in case of complications. 

Some unexpected findings of these patient preference surveys are in the population of COVID-19 vaccinated patients. In this population of elective spine patients, it seems that despite having gotten vaccinated, they still expressed high levels of fear of contracting COVID-19. This suggests that those more fearful of COVID-19 had a stronger incentive to get vaccinated. However, this does raise the question of whether the vaccination did give them any peace of mind with respect to contracting COVID-19 in the hospital setting. Further research would be necessary on this. Another fascinating disparity amongst elective spine patients was the disparity between sexes. Nearly 65% of men preferred to have elective spine operations in a hospital setting. While this correlates with a lower fear of contracting COVID-19, it may say something about preferences between sexes regarding the perception of risk and the value of "backup" resources in case of complications. 

A limitation of the surveys is that they can only measure the influence of COVID-19 on the patient's multifactorial decision between the ASC and hospital. It was not feasible for the study team to be able to provide an individualized estimate of cost savings due to the different nature of the procedures patients would be undergoing. However, studies have shown that undergoing procedures in an ASC instead of a hospital can lead to savings exceeding $3,500 for patients with commercial insurance [15]. While the increased length of stay of hospitals compared to ASCs was mentioned to patients in the survey, its influence in the survey was not directly measured. It is worth noting that hospital admission lengths for lumbar spine procedures average roughly 2.5 days, as compared to ASCs that almost always discharge their patients on the same day [16]. However, this difference is less significant due to the patients who undergo their procedures in hospitals typically being older, sicker, and undergoing more complex operations that require more time under observation. 

While the data in the surveys is interesting, the true value of these surveys lies in the patient's education and subsequent conversation with the surgeon. When patients completed this survey, they were introduced to the possible option for elective spine surgery to take place in different settings. They were also exposed to different benefits and drawbacks of each setting. The conversation regarding the surgical site (ASC or hospital) is one that happens in a spine surgeon's office multiple times per day. With these surveys, half of the work of educating the patient is done before the surgeon walks into the room. Patients have time to formulate more advanced questions, and surgeons can spend more time on meaningful interactions rather than repetitive regurgitation of the same basic information. The survey does not stand in place of a conversation between surgeon and patient but rather serves as a helpful tool to prime more effective and efficient conversation tailored to the patient's individual needs and concerns. For example, a patient may be fearful of contracting COVID-19 in a hospital setting and prefer an elective spine surgery to be performed at an ambulatory surgery center. If the surgeon feels the patient would be better suited to a hospital setting due to high risk or any other reason, a simple conversation can be had to explain the lengths taken to mitigate the spread of the disease in the hospital setting, like masks and frequent sanitization.

Alternatively, some patients may initially lean toward a hospital setting for concern over complications at an ASC. Concern given the out-of-hospital setting of ASCs is understandable. However, a systematic review has shown procedures being performed in hospitals have significantly higher rates of intraoperative events, postoperative complications, readmissions, and reoperations [16]. Using this information, surgeons can offer the concerned patient further education on how complications at an ASC are handled and reassure that carefully selected and planned operations are very safe to take place in an ASC. 

The differences in preferences for surgery location by gender may be partly explained by fear of COVID, but the lack of patient education on the difference between the ASC and hospital has been documented in other literature. Studies have suggested that patients do not have readily attainable literature to educate themselves on the differences between having procedures performed in ASCs and hospitals [17]. It is of paramount importance to educate patients on the different options they have for elective spine surgery locations, as well as the pros and cons of each location. This survey acts as a low-risk, high-reward tool to educate patients about their options and facilitate productive surgeon-patient conversations. Overall, this study serves to act as a proof of concept for optimizing surgeon-patient interactions regarding surgical locations in the context of elective spine surgery during and after the COVID-19 pandemic.

Limitations

Limitations of this study include sample size, geographical location of the sample, as well the level of inclusive demographic information. The patient population of 58 is largely composed of patients in Northern New Jersey, which is not suggestive of national or international populations. Due to the need for the survey to be short, anonymous, and to minimize hassle for patients and surgeons, there was a limited amount of information that could reasonably be collected in terms of patient demographics and risk factors. Another limitation of this study is that it did not follow patients to assess the outcomes of surgical interventions in the ambulatory or hospital-based settings. Further studies should look into preferences as COVID subsides in larger groups, as well as long-term outcomes of elective spine surgery in either setting.

## Conclusions

The results of the surveys showed the expected result that those with high fear of contracting COVID-19 were averse to having their procedures performed in the hospital by showing a strong preference for undergoing procedures in the ambulatory surgical center. Additionally, a strong preference for undergoing procedures in the hospital was seen in the male gender. The preference of males relative to females for undergoing procedures in the hospital could not be explained by the results of the survey. The difference in preferences by gender could be due to different perceptions regarding the risk and value of "backup" resources in case of complications. The findings of this survey highlight the need for patient education in regard to the safety of procedures being performed at an ambulatory surgical center, as well as the steps taken to mitigate the spread of COVID-19 and other infections in the hospital setting.
